# Comparison among the microbial communities in the lake, lake wetland, and estuary sediments of a plain river network

**DOI:** 10.1002/mbo3.644

**Published:** 2018-06-10

**Authors:** Wei Huang, Xing Chen, Kun Wang, Junyi Chen, Binghui Zheng, Xia Jiang

**Affiliations:** ^1^ National Engineering Laboratory for Lake Pollution Control and Ecological Restoration Chinese Research Academy of Environmental Sciences Beijing China; ^2^ State Key Laboratory of Environmental Criteria and Risk Assessment Chinese Research Academy of Environmental Sciences Beijing China

**Keywords:** community, estuary, lake, sediment, wetland

## Abstract

Sediment microbial communities from plain river networks exert different effects on pollutant transformation and migration in lake basins. In this study, we examined millions of Illumina reads (16S rRNA gene amplicons) to compare lake, lake wetland, and estuary bacterial communities through a technically consistent approach. Results showed that bacterial communities in the sampled lake sediments had the highest alpha‐diversity (Group B), than in sampled lake wetland sediments and estuary sediments. Proteobacteria was the most abundant (more than 30%) phyla in all the sediments. The lake sediments had more Nitrospirae (1.63%–11.75%) and Acidobacteria (3.46%–10.21%) than the lake wetland and estuary sediments, and estuary sediments had a greater abundance of the phylum Firmicutes (mean of 22.30%). Statistical analysis (LEfSe) revealed that lake wetland sediments contained greater abundances of the class Anaerolineaceae, orders Xanthomonadales, Pseudomonadales, and genera *Flavobacterium*,* Acinetobacter*. The lake sediments had a distinct community of diverse primary producers, such as phylum Acidobacteria, order Ignavibacteriales, and families Nitrospiraceae, Hydrogenophilaceae. Total phosphorus and organic matter were the main factors influencing the bacterial communities in sediments from several parts of the lake wetland and river estuary (*p* < .05). The novel insights into basin pollution control in plain river networks may be obtained from microbial distribution in sediments from different basin regions.

## 1 INTRODUCTION

Sediments are distinct components of basin ecosystems (Wang et al., 2012), and species and microbial diversity in sediments are considerably higher than those in water bodies (Zinger et al., 2011). As a main component of aquatic systems, sediment plays a critical role as nutrient provider, pool, and source (Huang et al., 2015). In a plain river network region, several types of sediment exist in different regions, such as lakes, wetlands, and estuaries. As sediment microbes from these regions affect entire aquatic systems (Shimeta & Cook, 2011; Shtarkman et al., 2013), understanding microbial communities in different basin regions is crucial.

Lakes, lake wetlands, and estuaries are the main components of a plain river network, and sediments in these regions are the media for matter transformation and migration. A lake wetland is a landform along the lake region and can be regarded as a filtration system of lake outflow. Bacterial communities in lake wetlands are ubiquitous and play key roles in ecosystem functions, including cycling of biologically active elements (Newton, Jones, Eiler, McMahon, & Bertilsson, 2011; Van der Gucht et al., 2007; Woese, Kandler, & Wheelis, 1990). Wetland ecosystems are the most biologically diverse around lake basins (Ding et al., 2015; Iasur‐Kruh, Hadar, Milstein, Gasith, & Minz, 2010) and provide habitats for biota (Wu et al., 2015). As main components of lake basins, estuaries are buffer zones between rivers and lakes or oceans; runoff particles and suspended sediments are transported, stored, and modified in estuaries (Arzayus & Canuel, 2005; Sun, Wang, Wu, Wang, & Li, 2011). Inorganic and organic nutrients are usually stored by sedimentation and released back to the water column, and microbial communities in sediments play important roles in substance export, regeneration, and biochemical cycling in estuaries (Pinckney, Paerl, Tester, & Richardson, 2001; Yokokawa & Nagata, 2010).

Previous studies separately analyzed microbial communities in lake, wetland, and estuary sediments. In particular, the correlation between microbial communities in wetlands and environmental factors were mostly investigated under horizontal gradients, such as C or N availability (Lin et al., 2012; Mackelprang et al., 2011; Moseman‐Valtierra, Armaiz‐Nolla, & Levin, 2010), temperature (Redmond & Valentine, 2012), pH (Lindstrom, Kamst‐Van Agterveld, & Zwart, 2005), and sediment structural characteristics (Liu, Ding, Jia, & Cai, 2012). The distribution of microbial communities in sediments, influence of microbial communities on nutrient or biogeochemical cycling in lakes (Cotner & Biddanda, 2002; Fang et al., 2015; Ligi et al., 2014; Song, Li, Du, Wang, & Ding, 2012), and interactions between microorganisms and sediment biogeochemistry in estuaries are researched independently (Freitag, Chang, & Prosser, 2006; Pimenov et al., 2010). Despite the importance of sediment microbes in distribution, substance cycling, and environmental influence in their respective regions, research on the overall distribution and differences in microbial community in sediments from a basin is limited. In response, the goal of this research was to expand the knowledge of microbial distribution in sediments from different basin regions of a plain river network. This would improve our understanding of microbial function in basin pollution control.

**Figure 1:**
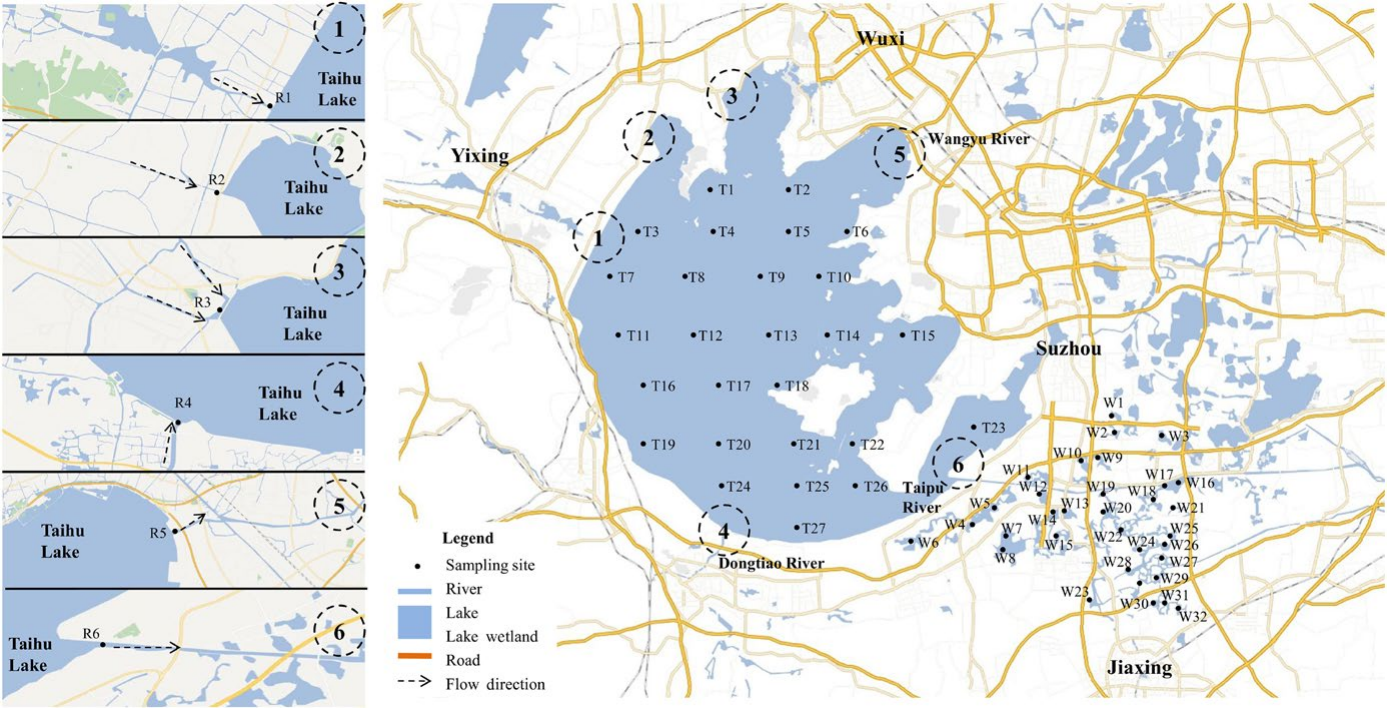
Sampling site at the study area

As one of the most densely populated regions in China, Taihu Basin (with a surface area of 1.58 × 10^4^ km^2^) is located in a typical plain river network in the middle‐lower Yangtze River plain of eastern China. Rivers and lake wetlands typically exist in Taihu Basin. Approximately, 20 large‐, medium‐, and small‐sized cities are located around Taihu Lake and have large inflow and outflow rivers, such as Taipu River, Wangyu River, and Dongtiao River, which are connected to Taihu Lake. The northwest part of Taihu Lake generally has small inflow rivers, and most of the pollutants from Yixing City and Changzhou City are discharged into Taihu Lake through these inflow rivers. Taihu Basin also has numerous lake wetlands, which are located in the eastern and southeast parts of Taihu Basin. Numerous lake wetlands exist around the Taipu River and influence the water quality in Jiaxing City. The location of Taihu Lake, the lake wetlands, and estuary rivers are shown in Figure 1.

In this study, we obtained millions of tags from the lake, lake wetland, and estuary sediments obtained from Taihu basin and compared the microbial communities in the sediments. We believe that this study can provide novel insights into basin pollution control in plain river networks.

## 2 EXPERIMENTAL PROCEDURES

### 2.1 Sediment collection and characterization

**Figure 2:**
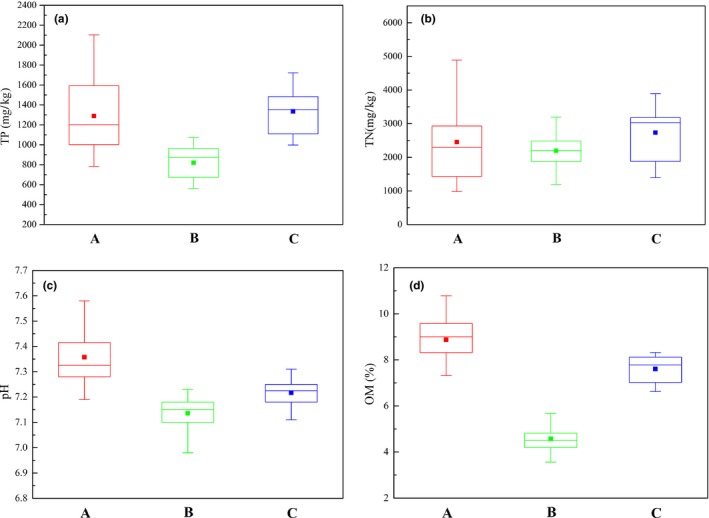
Physicochemical properties of the sediments from the three regions, Red (a) indicates the lake wetland, green (b) for the lake, and blue (c) for the river estuary. The horizontal lines composing the box from top to bottom represent 3rd quartile, median, and 1st quartile, respectively. The dot represents the average value

Taihu Lake is the third largest freshwater lake in China and located in the eastern plain of the country (30°55′40″–30°55′40″ N, 119°52′32″–120°36′10″ E) (Xu et al., 2015; Zhu et al., 2013). A total of 65 sediment samples were collected, 32 from the wetland in the southeast part of Taihu Lake (Group A), 27 from the main lake district (Group B), and six from the main river estuaries (Group C). Figure 1 shows the sampling sites in the three regions. The sediment samples were collected in July 2017, and the lake, lake wetland, and estuary sediments were assigned as T, W, and R, respectively. The weights of the collected samples were sufficient for DNA extraction and analysis of physicochemical parameters. After sampling, the sediment samples were placed in sealed plastic bags, stored in a portable ice box, transferred into the laboratory within 24 hr, and stored at −80°C before analysis. Each type of sediments was mixed with deionized water or 0.01 mol L^−1^ KCl solution in a 1:2.5 (w/v) mixture for pH measurement, and organic matter (OM) content in each sediment was calculated according to the loss on ignition to constant mass (4 hr) at 550°C (Huang et al., 2015). Total nitrogen (TN) and total phosphorus (TP) in sediments were measured with standardized methods and tests (Huang, Chen, Jiang, & Zheng, 2017). Physicochemical parameters are shown in Figure 2.

### 2.2 Extraction of genomic DNA

Total genomic DNA was extracted from the sediment samples using a PowerSoil DNA Isolation Kit (Mobio Laboratories Inc., San Diego, CA, USA) according to the manufacturer's protocol. DNA concentration and quality were determined using a NanoDrop Spectrophotometer (Thermo Scientific NanoDrop Lite, CA, USA). DNA was diluted to 10 ng μl^−1^ using sterile ultrapure water and stored at −80°C for further analysis.

### 2.3 PCR amplification

The V4–V5 regions of bacterial 16SrRNA genes were amplified with universal primers 515F (GTGCCAGCMGCCGCGGTAA) and 926R (CCGTCAATTCMTTTRAGTTT). These primers were selected because of their high coverage across phyla (Liu, DeSantis, Andersen, & Knight, 2008; Wang & Qian, 2009; Xiong et al., 2014). The PCR mixture (25 μl) contained 1 × PCR buffer, 1.5 mmol L^−1^ MgCl_2_, 0.4 μmol L^−1^ each of deoxynucleoside triphosphate, 1.0 μmol L^−1^ of each primer, 0.5 U of TaKaRa*Ex Taq*, and 10 mg of template DNA. The PCR amplification program was as follows: initial denaturation at 94°C for 1 min, followed by 30 cycles (denaturation at 94°C for 20 s, annealing at 56°C for 30 s, and elongation at 72°C for 45 s), and a final extension at 72°C for 5 min. Three replicates of PCR reactions for each sample were combined. A similar volume of 1× loading buffer (containing SYBR green) was mixed with PCR products and then subjected to electrophoresis on 2% agarose gel for detection of PCR product size. Samples showing a bright main strip of 300–500 bp were selected for further experiments. PCR products were purified with an OMEGA gel extraction kit (Omega Bio‐Tek, USA), and equal molar amounts of the products from different samples (65 samples from three different regions) were pooled. Sequencing libraries were generated with TruSeq DNA PCR‐free sample prep kit (Illumina) according to the manufacturer's recommendations, and index codes were added. Library quality was assessed on a Qubit^®^2.0 Fluorometer (Thermo Scientific, CA, USA) and Agilent Bioanalyzer 2100 system. The library was finally subjected to paired‐end sequencing (2 × 250 bp) on an Illumina Miseq apparatus from TinyGene Bio‐Tech Co., Ltd. (Shanghai).

### 2.4 Data analysis

The 16S sequences were analyzed using a combination of software mothur (version 1.33.3) (Schloss et al., 2009), UPARSE (Edgar, 2013), and R (version 3.2.3). The demultiplexed reads were clustered at 97% sequence identity into operational taxonomic units (OTUs) using the UPARSE pipeline (http://drive5.com/usearch/manual/uparse). The OTU representative sequences were assignment for taxonomy against Silva 119 database with confidence score ≥0.8 by the “classify.seqs” command in mothur. OTU's were named to the species level using SILVA taxonomic nomenclature.

Principal component analysis (PCA) was employed to explore and visualize the similarities between sediment samples obtained from three regions based on Bray–Curtis dissimilarity using package Ape (R package) (Paradis, Claude, & Strimmer, 2004). Redundancy analysis (RDA) based on population abundance and environment factors was performed using Canoco4.5. LEfSe (Linear discriminant analysis Effect Size) (Segata et al., 2011) was used to find indicator bacterial groups specific to the sediment samples (*p*‐value cutoffs .05). Independent *t*‐test and permutational multivariate analysis of variance (ggpubr package of R, *p*‐value .05) were used to identify the differences that exist among different groups.

### 2.5 Accession numbers

All of the sequencing data analyzed in this study can be downloaded from the NCBI's Sequence Read Archive using the accession numbers SRP133902 for samples from lake wetland (32 samples), lake (27 samples), and estuary (six samples).

## 3 RESULTS

### 3.1 Diversity indices of bacterial communities in the sediments from different regions

**Figure 3:**
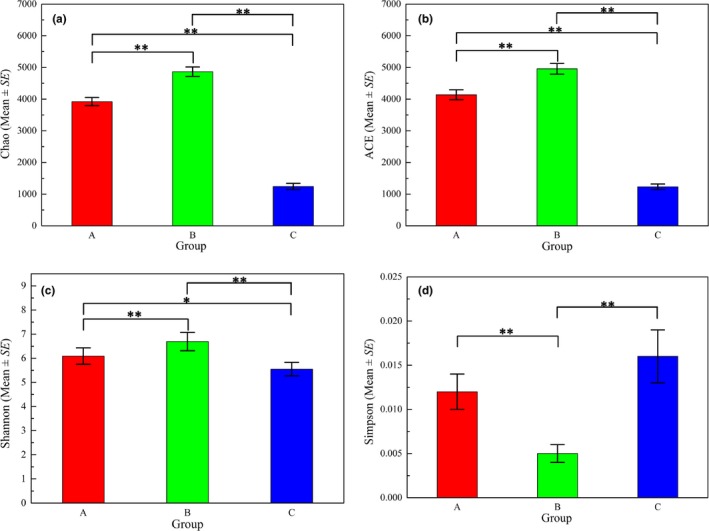
Diversity of the sediment samples from the three regions (a for the lake wetland, b for the lake, and c for the river estuary). *p < .05, *^*^ *p* < .01

A total of 65 sediment samples were obtained, 32 from the lake wetland (Group A), 27 from the lake (Group B), and six from the estuary (Group C). After tag merge and quality control, 2,822,582 reads were obtained. Although the total number of errors were low, stringent quality controls were set because of the overestimation of alpha‐diversity (Fang et al., 2015; Wang et al., 2012). Figure 3 shows the average values of the diversity indices (Chao, ACE, Shannon, and Simpson) and the results of variance analysis in the sediments from the three regions. The bacterial community in lake sediment had higher diversity than the lake wetland and estuary sediments. The Chao, ACE, and Shannon indices of the bacterial communities in all the sediments exhibited similar variation trend. The bacterial community from the lake had the highest Chao (4864.2 on average), ACE (4975.0 on average), and Shannon (6.69 on average) values, and lowest Simpson value (0.005 on average). The analysis of variance results indicated that the Chao, ACE, and Shannon values of the samples from the three groups varied significantly (*p* < .05) among each other. The Simpson index of sediment from the estuary showed the highest average value (0.016), and significant differences (*p*<0.01) existed between the samples from the lake and the lake wetland or the estuary.

### 3.2 Bacterial community composition in sediment samples

**Figure 4:**
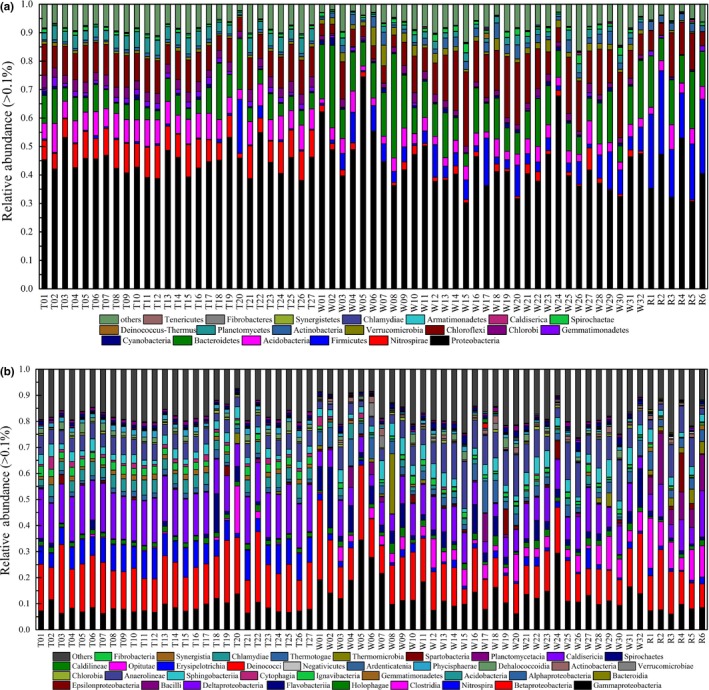
Relative abundances of different phyla (a) and classes (b) in the 65 sediment samples

The phylum and class level distributions of bacterial communities in the sediments are shown in Figure 4. A total of 34 phyla were detected in the sediment samples obtained from the three regions. Proteobacteria represented more than 30% of all phyla in sediments (Figure 4a), and Proteobacteria classes, such as Gammaproteobacteria, Betaproteobacteria, and Deltaproteobacteria, were the main classes in the sediments (Figure 4b). The relative abundance of Nitrospirae in the lake sediments was greater than those from the lake and estuary (8.53% ± 2.27% vs. 1.81% ± 1.42% or 0.24% ± 0.14%, respectively). Acidobacteria in the lake sediments had higher abundance than those in the lake wetland and inflow river (3.46%–10.21% vs. 1.49%–8.12% or 2.42%–8.73%, respectively). Firmicutes was more abundant (mean value of 22.30%) in estuary sediments than in lake (mean value of 1.32%) and wetland (mean value of 5.59%) sediments. Bacteroidetes (10.85%), Chloroflexi (11.09%), Cyanobacteria (0.62%), and Actinobacteria (1.94%) were the other highly abundant phyla in the sediment samples.

Figure 4b shows the changes in the bacterial community composition at the class level. Gammaproteobacteria, Betaproteobacteria, Nitrospira, Flavobacteriia, Deltaproteobacteria, and Holophagae were abundant in the three regions. Gammaproteobacteria was more abundant in the sediment from the lake wetland (14.27%) than in the sediments from the lake and the estuary (8.35% and 7.89%, respectively). *Clostridia* was more abundant in the wetland and estuary sediments (4.30% and 11.23%, respectively) than in lake sediments (1.13%). The lake sediments had more Nitrospira (8.53%) than lake wetland and estuary sediments (1.81% and 1.53%, respectively). Nitrospira and Deltaproteobacteria were more abundant in the lake sediments than in the wetland and estuary sediments (*p* < .01). Bacilli, Epsilonproteobacteria, Gemmatimonadetes, Ignavibacteria, Chlorobia, and Phycisphaerae were the other classes detected in the sediment samples from the three regions.

**Figure 5:**
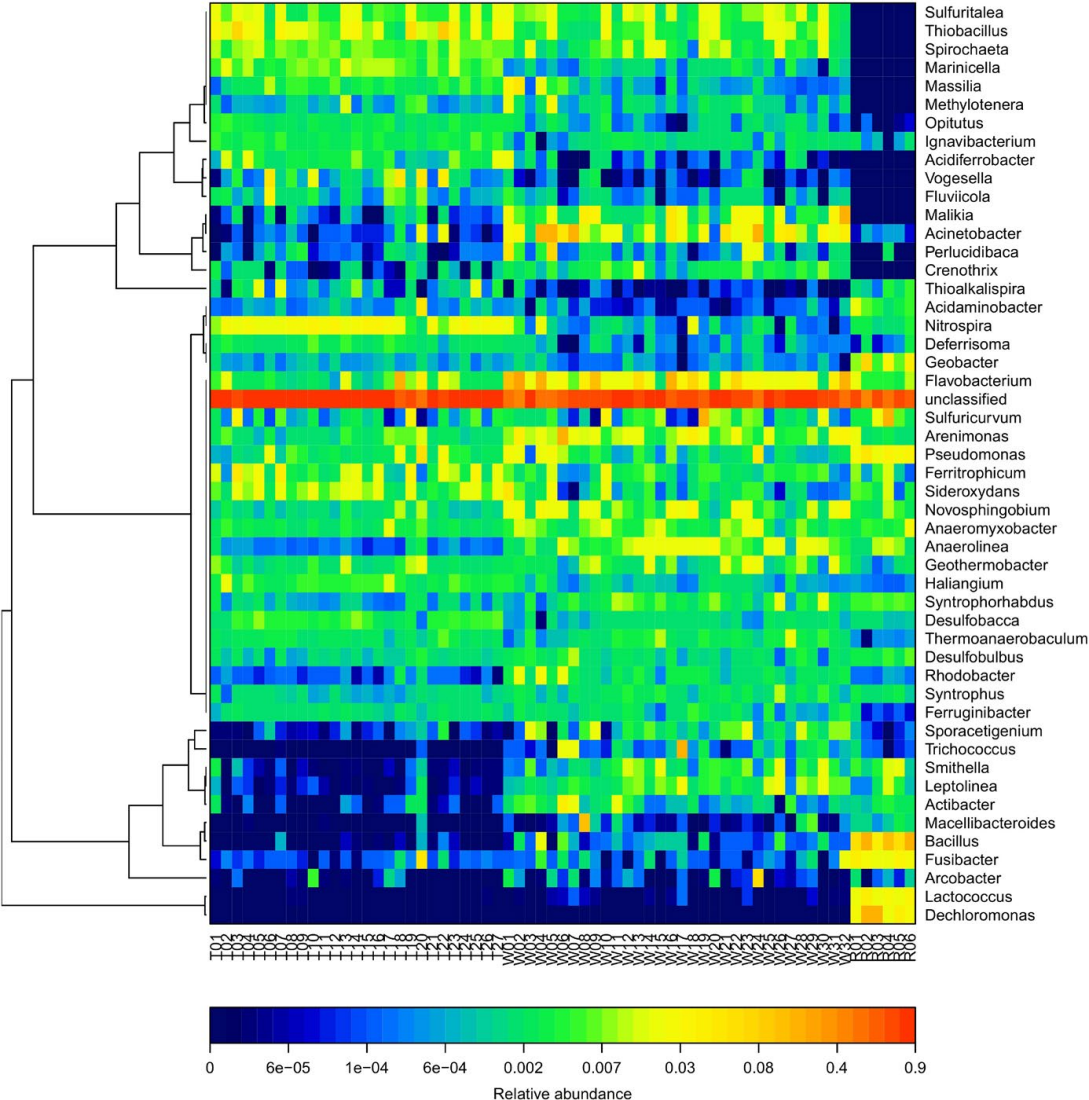
Heatmap analysis of bacterial communities at the genus level

The taxonomic composition at the genus level of the bacterial communities in sediment samples from the three regions is shown in Figure 5. *Nitrospira*,* Thiobacillus*, and *Sulfuritalea* were the most dominant genera in most of the sediment samples from the lake region with relative abundance of more than 4%. The bacterial communities in the wetland sediments had relatively higher abundance of *Flavobacterium* and *Acinetobacter* than those in the lake and estuary sediments. The relative abundance of *Bacillus*,* Fusibacter*,* Lactococcus*, and *Pseudomonas* in the sediment samples from the estuary was more than 6%, which was higher than those in the sediment samples from the two other regions (lower than 1%). *Thiobacillus*,* Anaerolinea*,* Desulfobulbus*,* Rhodobacter*, and *Novosphingobium* were the other dominant genera with relatively high abundance in the sediment samples from the three regions.

### 3.3 Principal component analysis

**Figure 6:**
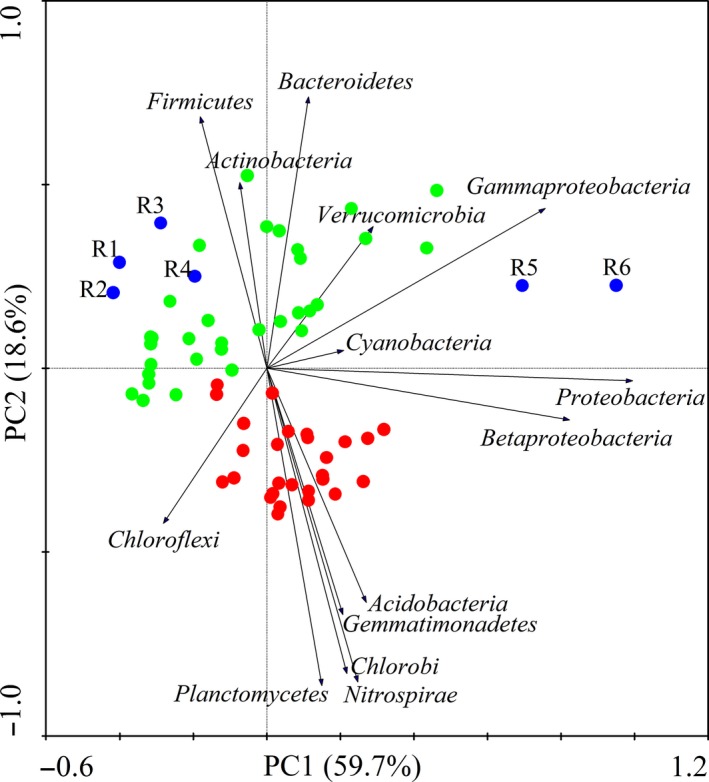
PCA of phylum abundance data using Canoco 4.5 (red represents the lake, green represents the lake wetland, and blue represents the estuary)

Figure 6 shows the groupings of the sediment samples according to their bacterial community structure. Two PCAs explained 78.3% of the total variation in the microbial community structure. The result indicated that the sediments obtained from the three regions had significantly different bacterial phyla. Acidobacteria (7.53%), Gemmatimonadetes (1.56%), Chlorobi (3.39%), Nitrospirae (8.53%), and Planctomycetes (4.18%) were abundant in the lake sediments, while Firmicutes (5.59% and 2.23%), Actinobacteria (2.65% and 2.58%), Bacteroidetes (13.29% and 13.91%), and two Proteobacteria groups, Gammaproteobacteria (14.27% and 7.89%) and Betaproteobacteria (14.81% and 14.11%), were abundant in the wetland and estuary sediments. The sediments from R5 and R6 had more Cyanobacteria and Verrucomicrobia abundance than the sediments from other parts of the estuary.

### 3.4 Redundancy analysis

**Figure 7:**
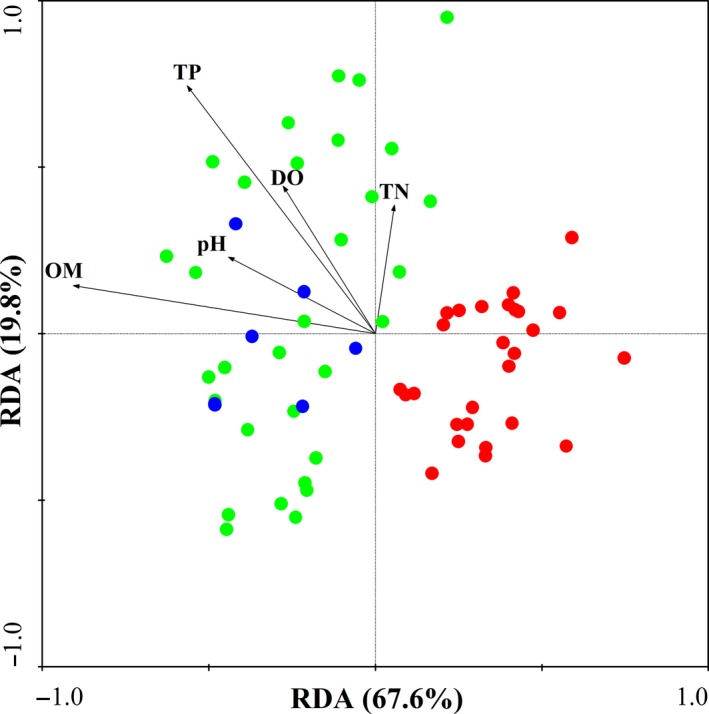
RDA of the bacterial communities as affected by environmental conditions and based on the relative abundance of dominant bacterial phyla (red represents the lake, green represents the lake wetland, and blue represents the estuary)

RDA was performed on the basis of the abundance at the phylum level and environmental factors (physicochemical parameters). Figure 7 shows the relationship between bacterial community composition and physicochemical parameters. The first axis explained 67.6% of the bacterial diversity, whereas the second axis explained 19.8% of the variation. Few physicochemical parameters influenced the bacterial community in the sediment from the lake, and TN influenced the bacterial community in the sediment to a certain extent. The bacterial communities in the sediments from the lake wetland and estuary were associated with physicochemical parameters, such as OM, pH, DO, and TP content. The bacterial communities in the sediments from several parts of the lake wetland and the river estuary were positively correlated with TP and OM (0.894, *p* < .05).

### 3.5 LEfSe analysis based on community abundance

**Figure 8:**
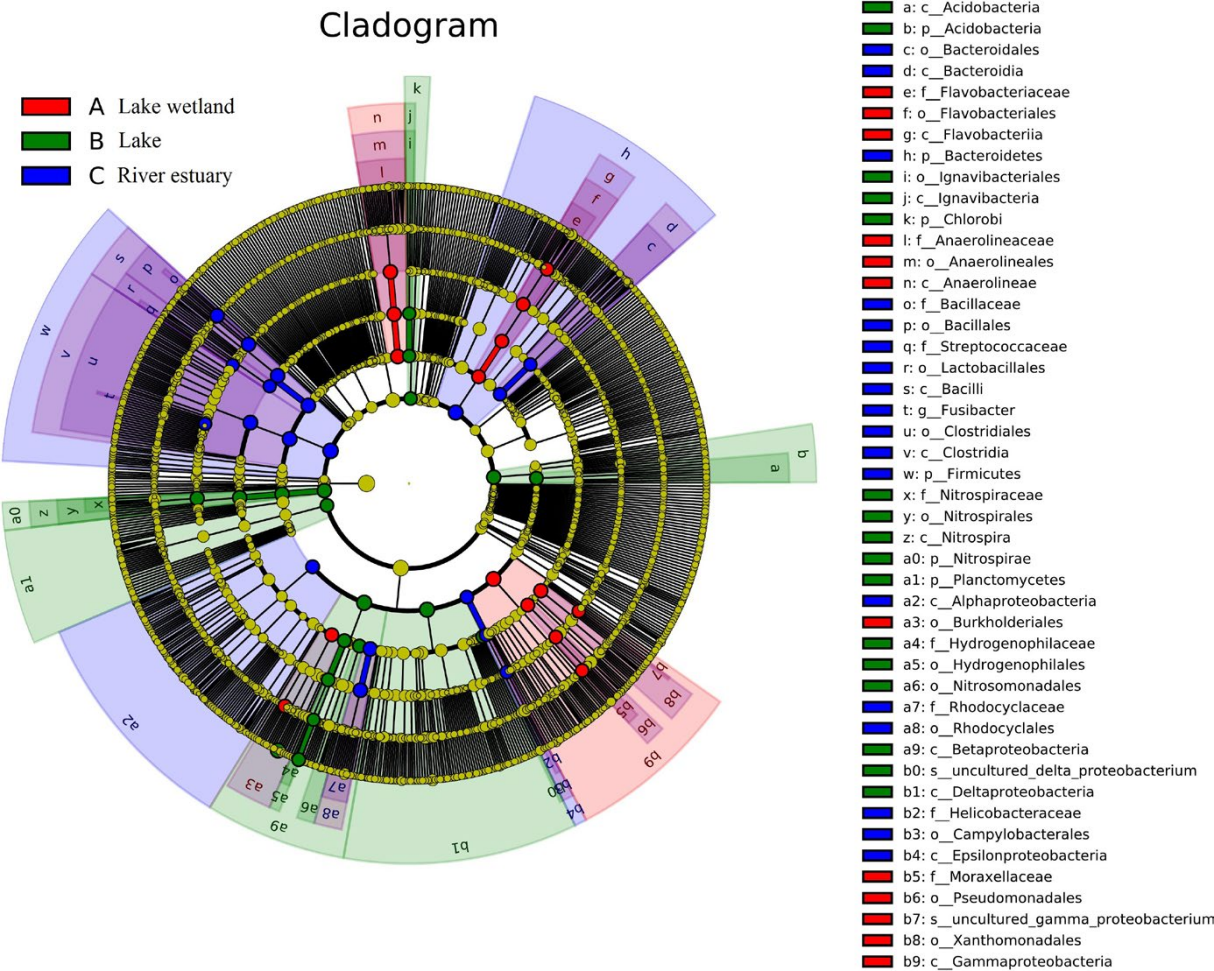
Cladogram showing the phylogenetic distribution of microbial communities associated with the sediments from the three regions; lineages with LDA values of 3.0 or higher as determined by LEfSe are shown. Differences are represented by the color of the most abundant class. Red (a) indicates lake wetland region, green (b) lake region, and blue (c) river estuary region; yellow represents insignificant difference. The diameter of each circle is proportional to a taxon's abundance. Circles from inner region to outer region represent the phylogenetic levels from domain to genus

**Figure 9:**
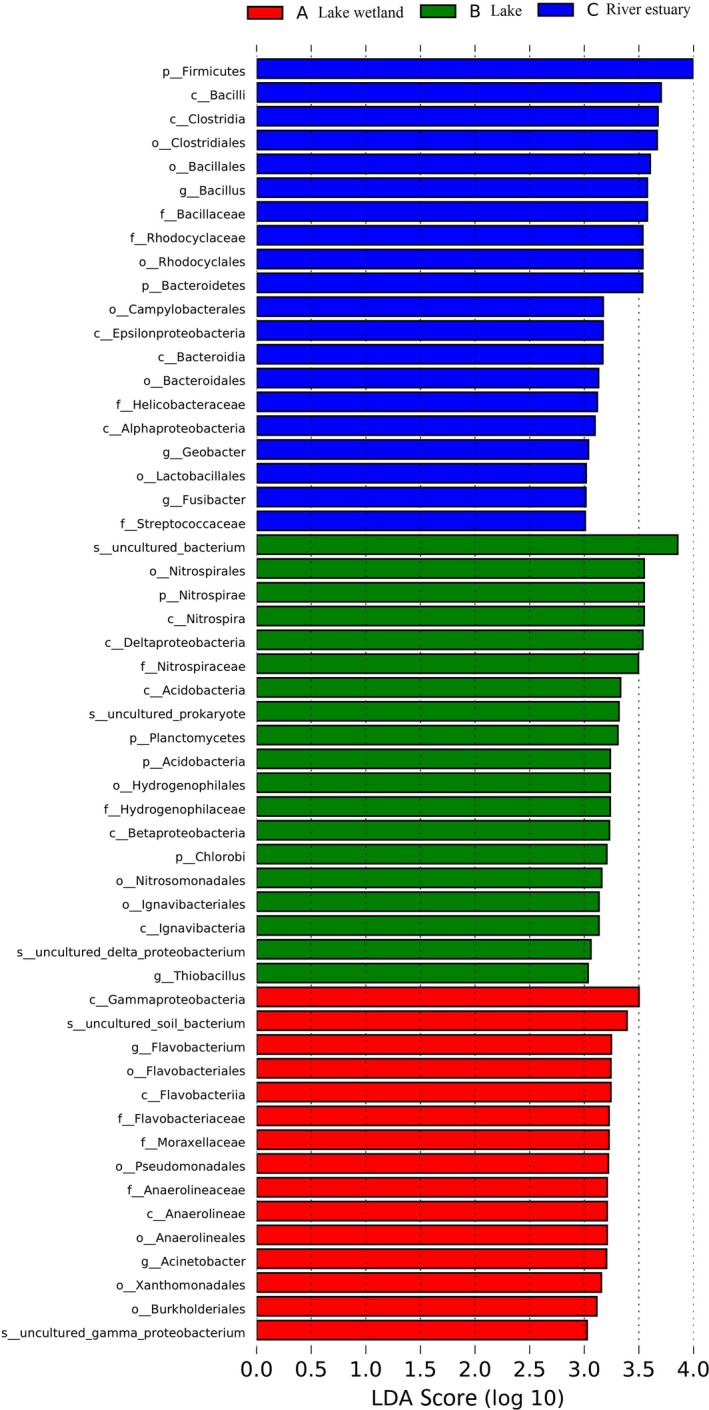
Indicator microbial groups in the three groups of sediment samples with LDA values higher than 3.0

Figure 8 shows that the bacterial communities dominant in the lake wetland were Flavobacteria (the class and order of Flavobacteriales), Anaerolineae (the class and order of Anaerolineales), and Gammaproteobacteria (the class and orders of Xanthomonadales and Pseudomonadales). The lake wetland had LDA values greater than 3.0 for *Flavobacterium,* Pseudomonadales*,* Anaerolineaceae*, Acinetobacter, and* Xanthomonadales (Figure 9).

Five bacterial taxons were abundant in the lake sediments (Figure 8), namely, Nitrospirae (from phylum to family), Acidobacteria (from phylum to order), Chlorobi (from phylum to family), Deltaproteobacteria, and Betaproteobacteria (the class and orders of Hydrogenophilales and Nitrosomonadales).The lake sediment only had Nitrospiraceae, Acidobacteria, Hydrogenophilaceae, and Ignavibacteriales taxons exceeding LDA values greater than 3.0 (Figure 9).

The Firmicutes and Bacteroidetes *phyla* were abundant in the estuary sediments (Figures 8 and 9), particularly Bacilli, Clostridia, and Bacteroidia. The bacterial communities abundant in the estuary were Rhodocyclales (an order within Betaproteobacteria) and Epsilonproteobacteria. Among these bacterial communities, several fine communities, namely, *Bacillus*, Clostridiales, Bacillaceae, Rhodocyclaceae, Rhodocyclales, Campylobacterales, Helicobacteraceae, *Geobacter*, and *Fusibacter*, had an LDA value of 3.0 or higher.

## 4 DISCUSSION

Different regions, such as wetland, estuary, and lake side zone, exist in the Taihu Basin. The sediments in these regions are critical to pollutant release and can influence the water quality in Taihu Lake. The sediment samples from the lake wetland, lake, and river estuary were collected, and the differences among the microbial communities in the three regions were studied.

The diversity indices revealed that the Chao, ACE, Shannon, and Simpson values of the samples from the three regions varied significantly. This result indicated that the bacterial communities in the sediments from the three regions had significant regional differences. Numerous inflow and outflow rivers exist in Taihu Basin. Rivers 1–4 (Figure 1) are the main inflow rivers of Taihu Lake, and Rivers 5 and 6 are the outflow rivers. Pollutants entered the lake region through the inflow rivers and were discharged through the outflow rivers. Large pollutants deposition occurred in lake region, which caused the bacterial communities in the sediment from lake region to have higher diversity than those in the sediments from the two other regions (Deng, Cui, Hernandez, & Dumont, 2014; Liu et al., 2009). The bacterial community in the sediment from the lake wetland also had relatively high diversity because of the high amount of pollutant discharge from cities such as Suzhou and Jiaxing (Figure 1). Although the pollution source exists around the inflow and outflow rivers, most of the pollutants do not easily deposit because of the high‐flow velocity. The bacterial diversity in the sediments from the four inflow rivers (Rivers 1–4) was therefore lower than those in sediments from the two other regions (Beardsley, Moss, Malfatti, & Azam, 2011; Wu et al., 2017). In addition, as shown in Figure 2, the sediments from the outflow rivers had low pollutant concentrations, and the sediments from outflow rivers also had the lowest bacterial diversity.

Proteobacteria, a phylum that commonly exists in sediment, is nearly the most abundant phylum detected in samples among the three regions and plays a critical role in degradation and metabolism (Bai et al., 2012; Chaudhry, Rehman, Mishra, Chauhan, & Nautiyal, 2012; Huang & Jiang, 2016). Studies revealed that abundance of Acidobacteria is significantly correlated with pH, and Acidobacteria prefers an environment with low pH of ~5.5 (Jones et al., 2009). The sediment from the lake had the relatively lowest pH, and Acidobacteria was abundant in the sediment samples from the lake. The sediment from the lake also had higher Nitrospirae content than those in the sediments from the two other regions. In Taihu Basin, the lake region had high nitrogen concentration (2.62 ± 1.46 mg L^−1^, 2013–2014) (Wu, Qin, Yu, Deng, & Zhou, 2016), and the nitrogen release in sediment contributed to the high nitrogen concentration in the lake. Nitrospirae participates in nitrogen cycling and may be one of the main reasons for the high nitrate concentration in the sediments (Huang et al., 2017). The result also indicated that nitrogen might be the factor limiting eutrophication, and our previous study obtained similar results (Huang et al., 2017). The bacterial distribution at the genus level also indicated that the NO_3_–N concentration was higher in the lake than in lake wetland and river estuary because of the participation of *Thiobacillus* in NO_3_–N metabolism (Bruckner et al., 2013; Li, Zhao, & Wang, 2011). In the sediment from the estuary regions, the bacterial community from outflow river (R5 and R6) had higher abundance of Chloroflexi than that in other sediments from the inflow rivers. Chloroflexi, a photoautotrophic microbe, may have participated in organic pollutant degradation, thereby possibly causing the low OM content in the outflow rivers. The lake wetlands, located in the southeast part of Taihu basin, are polluted with agricultural pollutants and industrial pollution discharge (Beardsley et al., 2011; Bissett, Bowman, & Burke, 2006) from Jiaxing City (Figure 1). The region has more organic pollutants than the lake and estuary, especially in sediments. As a result, the lake wetland sediments had high abundance of Gammaproteobacteria, which are usually present in organic‐rich sediments (Figure 4b) (Song et al., 2012).

Taihu Lake is an algae‐dominated area, and algal blooms typically occur in summer. The algal growth and metabolism are significantly related to the sediment. Previous studies revealed that P is a major limiting factor of eutrophication in Taihu Lake, and the sediments of the algae‐dominated area is dominated by *Microcystis* (Huang et al., 2017; Shao et al., 2011; Wu, Chen, Xu, Liu, & Hahn, 2007). The results of this study revealed that TN concentration in water and sediment significantly influences bacterial community, and *Nitrospira* (phylum Nitrospirae) and *Thiobacillus* (phylum Proteobacteria) are significantly related to TN concentration. These two genera were also abundant in the lake sediments (Figure 5). N forms and concentrations were affected by Nitrospirae, and *Thiobacillus* directly participated in NO_3_–N metabolism and subsequently influenced TN concentration in the lake (Bruckner et al., 2013; Li et al., 2011). Therefore, TN concentration in sediment is possibly one of the main factors limiting eutrophication, and the limiting factor of eutrophication in Taihu Lake may be changed from P to N.

In the lake wetland around Taihu Lake, a large amount of pollutants from the towns and cities entered the rivers, which are connected with wetlands, and organic matters were deposited into this region, which caused the high OM content in the lake wetland region. Nutrients in this region are frequently transformed from inorganic to organic forms, and may explain the association between Desulfarculaceae (phylum Proteobacteria and class Deltaproteobacteria) and OM. The results indicated that the bacterial communities in sediments from parts of the lake wetland were influenced by TP and OM, and Proteobacteria was significantly positively correlated with TP and OM (*p* < .05). The inorganic P might therefore transform to organic P, and Desulfarculaceae might be one of the main OM indicators in the lake wetland (Jorgensen, 1982; Pallud & Van Cappellen, 2006).

The sediment samples from R1 to R4 are located in the estuary of inflow rivers. Large amounts of pollutants mixed, and parts of the pollutants were deposited into the surface sediments (Liu et al., 2009). The bacterial communities were influenced by the OM, TP, pH, and DO, indicating that the environmental conditions in this region were complex, and the bacterial communities were affected by these factors. In addition, R5 and R6 are the estuaries of outflow rivers. In these regions, flow velocities are high, and pollutants frequently migrated and transformed (Bai et al., 2009). Pollutants, such as inorganic and organic matters, are hardly deposited into the sediments. OM, DO, and pH, are not the main environmental factors that influence bacterial communities in this region.

In summary, the bacterial communities in the lake, wetland, and estuary sediments varied significantly, and the bacterial community varied according to region. In this study, we comprehensively compared the three regions (i.e., lake, wetland, and estuary) in a plain river network. The lake sediments had the most diverse bacterial communities, followed by the wetland sediments. Proteobacteria was the most abundant of all phyla in the sediments from the three regions. Nitrospirae, Acidobacteria, and Firmicutes were the most abundant phyla in the lake, lake wetland, and estuary sediments, respectively. TN affected the bacterial community in sediments from the lake region, and physicochemical parameters, such as OM, pH, DO, and TP concentrations, influenced the bacterial community in the wetland sediments. TP and OM contents were positively correlated (*p* < .05) to the distinct bacterial communities in sediments from several parts of the estuary regions.

## ACKNOWLEDGMENT

This study was supported by the Major Science and Technology Program for Water Pollution Control and Treatment (No. 2017ZX07206), Beijing Natural Science Foundation (8174080), and the project was funded by China Postdoctoral Science Foundation (2017M610968).

## CONFLICT OF INTEREST

None declared.
